# Prediction and accuracy improvement of insulin pump in-fusion deviation based on LSTM and PID

**DOI:** 10.1371/journal.pone.0324261

**Published:** 2025-06-04

**Authors:** Leijie Wang, Xudong Guo, Qiuyue Peng, Hongmei Zhang, Yuan Yang, Hongyan Wang, Yongxin Wang, Haofang Liang, Wuyi Ming, Zhen Zhang

**Affiliations:** 1 School of Mechanical Engineering, Dongguan University of Technology, Dongguan, China; 2 Mechanical and Electrical Engineering Institute, Zhengzhou University of Light Industry, Zhengzhou, China; 3 Department Emergency of Internal Medicine, Hubei Provincial Hospital of Traditional Chinese Medicine, Wuhan, China; 4 Zhengzhou Phray Technology co., Ltd., Zhengzhou, China; 5 Laboratory of Regenerative Medicine in Sports Science, School of Sports Science, South China Normal University, Guangzhou, China; 6 Guangdong HUST Industrial Technology Research Institute, Guangdong Provincial Key Laboratory of Digital Manufacturing Equipment, Dongguan, China; 7 School of Aerospace Engineering, Huazhong University of Science and Technology, Wuhan, Hubei, China; King Fahd University of Petroleum & Minerals, SAUDI ARABIA

## Abstract

In order to further improve the injection precision of the PH300 insulin pump, this paper optimizes and improves the mechanical structure and control algorithm of the PH300. The improved PH300 uses a proportional-integral-derivative controller based on back propagation neural network (BP-PID) algorithm to control operation, and the experimental results show that the minimum effective single infusion dose of the improved PH300 is 0.047 U, which is reduced by 50.52%. The deviation reduction of low-dose infusion (0.1U-0.9U) ranged from 1.47% to 10.87%, with a mean of 4.91%. The mean deviation of the improved PH300 decreases by 12.85% after a 24h low basal rate (0.5U/h) injection. In addition, Long Short-Term Memory (LSTM) was used to predict the deviation during injection, and the predicted values were uniformly compensated for in subsequent injection experiments. The LSTM model performed best with a training set of 85%, a test set of 15%, an epoch of 300, a batch number of 256, and 32 hidden layer neurons. After compensation, the mean infusion deviation for large doses was reduced by 12.05%, and the maximum deviation by 14.12%.

## 1. Introduction

Diabetes mellitus is a metabolic disorder of sugars, proteins, fats, etc., caused by deficiencies in insulin secretion or insulin resistance [1–3]. In 2022, approximately 828 million adults (aged 18 years and older) were estimated to be living with diabetes, marking an increase of 630 million compared to 1990 (95% credible interval [CrI] 757–908 million) [4]. By 2050, the global prevalence is projected to exceed 1.31 billion (1.22–1.39 billion) [5]. As of 2020, there were approximately 400 million people living with diabetes worldwide, and 592 million are expected to be by 2035 [6]. Worldwide, approximately 1.6 million people die directly from diabetes each year [7], with healthcare spending on diabetes approximately reaching 490 billion dollars in 2025 [8]. More than 200 million people worldwide currently use exogenous insulin once or more times a day to treat diabetes [9]. Diabetes has emerged as a critical public health concern, with its uncontrollable progression potentially inflicting both direct and indirect harm to the healthcare system [10,11]. The use of insulin pumps for insulin infusion in vitro is one of the most important methods for the treatment of diabetes. Diabetes management has been transformed by insulin pumps, which provide a more precise and individualized insulin delivery method compared to conventional injection therapies [12]. The insulin pump simulates the secretion of physiological insulin in the human body to the greatest extent by setting the basal rate, temporary basal rate, and pre-meal high-dose infusion modes [13–15]. Infusion accuracy is one of the most important performance indicators of insulin pumps, which is the key to determining the performance of insulin pumps and is the key technology and bottleneck technology in the design.

Since the introduction of the first closed-loop system in the 1960s and 1970s, advancements in diabetes management have been closely tied to innovations in diabetes technology, particularly the widespread adoption of continuous insulin delivery systems and glucose monitoring devices [16,17]. Boiroux and Jørgensen [18] evaluated the performance of a nonlinear model predictive control algorithm for closed-loop insulin delivery in type 1 diabetes (T1D) management, with simulations demonstrating its safety and efficacy in optimizing insulin administration. Ozaslan et al. [19] further investigated the feasibility of a closed-loop insulin delivery system employing a zone model predictive control algorithm for pregnancies complicated by T1D, confirming its applicability in this patient population. Plunger insulin pumps have high precision and mature theory and application technology, such as those from Medtronic, Dana, and Roche [20–22]. Insulin pumps are designed to provide continuous basal insulin doses and variable bolus doses, which contribute to improving blood glucose control [23]. For insulin pump infusion, it is crucial to ensure both the accuracy of a single large dose infusion and continuous base rate infusion within a reasonable range. The higher the dose of single-needle infusion, the higher the accuracy of observation [24]. In the case of low-dose insulin injection and low basal rate infusion, frequent bubble formation can lead to poor treatment [25]. Thornton et al. [26] emphasized that the rapid initiation of infusion and accurate delivery at a low infusion rate are essential to ensure patient safety and maintain the integrity of feedback control. The injection accuracy of the plunger insulin pump depends mainly on the precise control of the position or speed of the brushless direct current (DC) motor. Vector control [27] and proportional integral differential [28] were used to optimize the control of the brushless DC motor. A parameter-adaptive PID control algorithm based on a genetic algorithm was proposed by Shen [29], effectively mitigating the limitations of traditional PID controller tuning, including low control accuracy and poor stability. Osgouie et al. [30] employed an adaptive fuzzy logic control algorithm for closed-loop insulin injection control in diabetic patients. The results indicated that adaptive fuzzy logic control could reduce blood glucose levels from 14 mmol/L to 4.5 mmol/L within three hours. However, due to the frequent start and stop braking of the motor during the use of the insulin pump, the motor control algorithm has difficulty solving the problem that the motor exceeds the predetermined angle due to inertia. Long short-term memory (LSTM) [31] is a variant of recurrent neural network (RNN) designed to alleviate the problem of gradient explosion or disappearance in RNN [32–35]. Yuan et al. [36] studied the application of the LSTM model in aero engine fault diagnosis and prediction. Xiao et al. [37] proposed a fault diagnosis method for three-phase asynchronous motors based on LSTM, which has the ability to learn meaningful representations from the original signals. An adaptive LSTM framework incorporating dual attention mechanisms was developed by Wang et al. [38] to automatically predict tool wear in smart manufacturing. Zhao et al. [39] conducted an empirical evaluation of the machine health monitoring system based on LSTM in the tool wear test. A hybrid Transformer-LSTM model was adopted by Bian et al. [40] to enhance the accuracy of future blood glucose level predictions using real-time data from continuous glucose monitoring systems. Mulerikkal et al. [41] used the space-time LSTM model to analyze and predict passenger flow from two dimensions of space-time. Zhang et al. [42] proposed that LSTM learn the long-term correlation between lithium-ion battery capacity and degradation factors to achieve accurate prediction of battery remaining life. In the field of blood glucose monitoring, Iacono et al. [43] proposed a hypoglycemia and hyperglycemia prediction alarm system based on personalized LSTM models. In addition, a hardware implementation of a low-power LSTM neural network for wearable medical devices was proposed by Tena et al. [44], with the goal of predicting blood glucose levels 30 minutes in advance. In summary, artificial neural network requires fixed-length inputs to process only static data, which cannot capture temporal correlations; LSTM can more accurately fit nonlinear features in the dynamic equilibrium of temporal data through memory cells and gating mechanisms (input/forget/output gates). Compared to RNNs, which are susceptible to noise interference, LSTM’s forgetting gate dynamically filters invalid historical information and retains key states.

In this study, the current commercialized PH300 insulin pump was used to improve the accuracy of single low-dose, low-base-rate insulin infusion by improving the mechanical structure of the insulin pump. There are limitations of traditional PID in dynamic control, such as the inability of fixed parameters to handle nonlinear variations or delay effects. Optimization of PID parameters using BP neural networks provides a basis for prospective adjustment and improvement of BP-PID. In this paper, the BP-PID system is utilized to dynamically control the injection mechanism of the pump, thus initially improving the injection accuracy. In addition, the prospective compensation of LSTM is employed to identify the direction of deviation in advance, which further improves the injection accuracy and reduces the insulin pump deviation. Relative to the existing studies, the innovation of this paper has two main points. On one hand, the internal structure of the insulin pump was optimized to reduce the minimum resolved amount of the single output flow of the pump and improve the pump refinement output capability in order to improve the accuracy of the personalized calculation of insulin injection rate. On the other hand, further improvement of insulin pump infusion accuracy was achieved by establishing an LSTM error prediction model for infusion compensation. With the aid of the innovative integration of hardware and software, the infusion accuracy of the insulin pump was improved overall.

## 2. Experimvental equipment and methods

### 2.1. Experiment equipment

**Fig 1:**
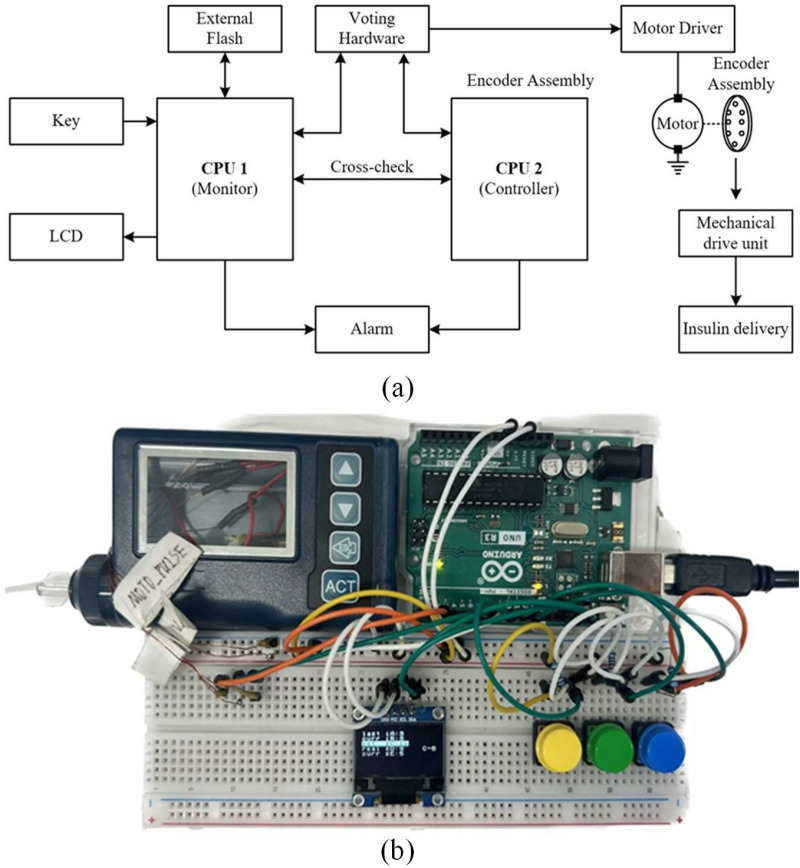
Workflow and structure of the insulin pump. (a) flow chart of insulin pump work structure. (b) PH 300 insulin pump experimental platform for algorithm development.

**Fig 2:**
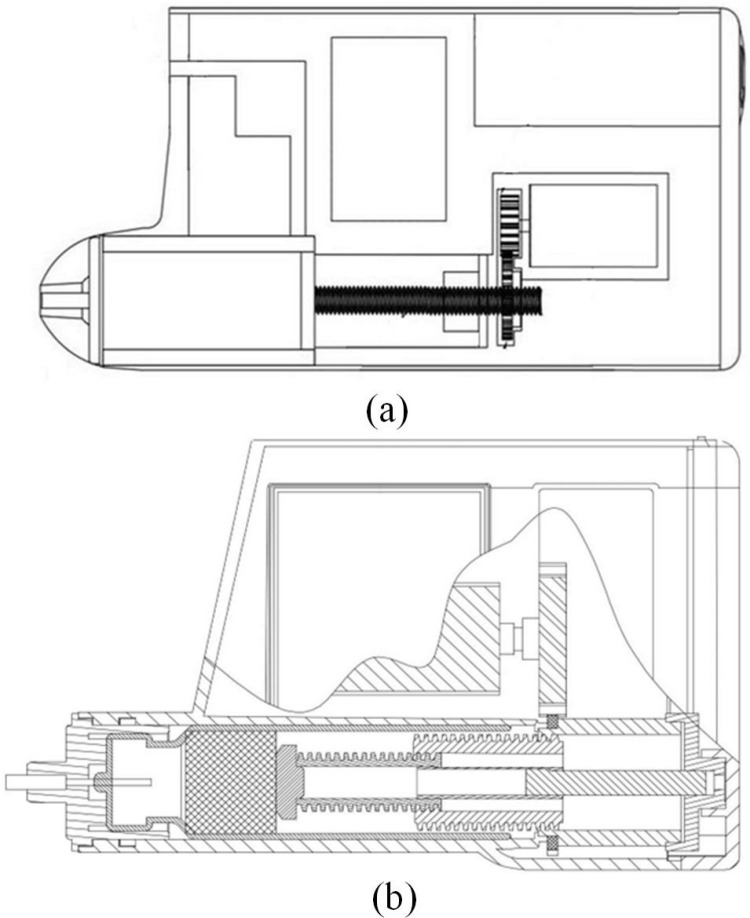
Comparison of the transmission structure of the insulin pump. (a) one-stage screw drive. (b) two-stage screw drive.

The insulin pump (PH 300) developed by Zhengzhou Phray Technology Co., Ltd. was adopted. The whole pump volume was 78 × 52 × 22 mm, and the weight was 55 g. The working principle and experimental platform of PH 300 are shown in Fig 1a and b. The PH 300 adopts a plunger transmission mechanism, which is widely used in insulin pump design, has good control performance, and has great application value in realizing precise drug delivery technology. Its main technical parameters are shown in S1 Table in S1 File. The insulin pump is a portable device, so it must have the characteristics of being small and lightweight [45,46]. The structure of the PH 300 insulin pump was improved in order to improve the accuracy of insulin infusion. The PH 300 insulin is placed in a user-replaceable needle that is placed inside the pump to form a special syringe with a piston. In this study, the one-stage screw was improved to a two-stage screw. The two-stage screw occupies 70% of the volume of the one-stage screw pump when it is not deployed, and the advancing stroke after deployment is about 1.67 times that of the one-stage screw. The internal structure of the one-stage and two-stage insulin pumps is compared as shown in Fig 2a and b.

### 2.2. Infusion delivery methods

The setting of the total amount of insulin in the pump is an extremely critical step that is related to the strength, speed, and safety of blood sugar control. The total amount of insulin is calculated as follows (for patients who have not received insulin therapy), listed in Eq (1): [47,48]


$$\begin{array}{c} U=\frac{6K\cdot (P-100)}{2000} \end{array}$$ (1)

where *U* represents the daily dosage of insulin (unit: mg); *P* represents the fasting blood glucose (unit: mg dL-1); *K* represents the patient weight (unit: Kg); 100 indicates the reference value of human normal blood glucose (unit: mg dL-1).

The total daily dose of insulin in diabetic patients is divided into a basal dose and a pre-meal high dose. The basal dose accounts for about 40%–60% of the total amount of insulin throughout the day, and the infusion period of the basal dose should also be set according to the specific situation of the patient’s blood sugar fluctuations and living conditions. There are many setting modes for basic infusion, and 1–24 times can be set according to the needs of blood sugar control [49]. In this study, the basic infusion was set at 40% of the total daily islet hormone, and the three-stage infusion method was adopted. The high dose before meals was set at 60% of the total daily insulin and distributed in a ratio of 1:1:1 before three meals. In this study, patients with a body weight of 75 Kg and fasting blood glucose of 220 mg dL-1 were used as references, and the infusion mode as shown in Table 1 was set for the experiment. In order to simplify the experimental process, the infusion accuracy of the insulin pump was verified by statistical deviation for each infusion of 2 U of insulin.

**Table 1:** Design of insulin infusion regimen for experimental verification.

Type	Dose	Time	Delivery method
Large dose before meal	4U	4:00-6:00	A single dose of 2 U, three times
Basal dose	5U	6:00-11:00	Infusion 0.1 U every 6 minutes
Large dose before meal	4U	11:00-13:00	A single dose of 2 U, three times
Basal dose	5U	13:00-18:00	Infusion 0.1 U every 6 minutes
Large dose before meal	4U	18:00-20:00	A single dose of 2 U, three times
Basal dose	5U	20:00-1:00	Infusion 0.1 U every 6 minutes

### 2.3. Experimental measurement methods

**Fig 3:**
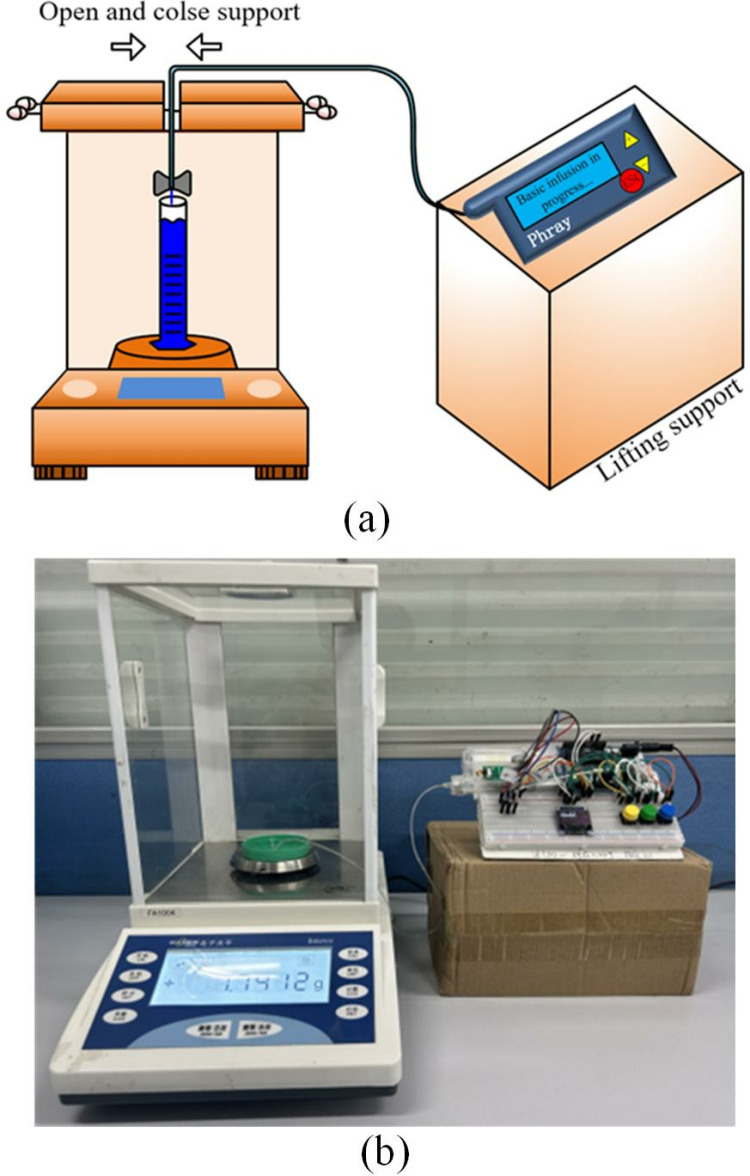
Schematic diagram of experimental setup for in vitro study. (a) model. (b) entity.

The typical fast-acting insulin used in in vitro insulin pump experiments is the conventional concentration (U-100), equivalent to 100 U mL-1. However, due to experimental constraints, distilled water was employed as a simulation for insulin injection in this study. Consequently, the weight of a 0.1U insulin injection should be 0.001g. Following the IEC 60601-2-24 micro weight method [50], a continuous weighing method was employed to measure the injection weight of insulin. For each infusion experiment, the measurement was repeated 6 times after each infusion, and the mean value was taken as the actual value of the insulin infusion dose. Actual measurement values and deviations could be recorded at different nodes as required by the experimental design. The test bench was located in a controlled room with a temperature of 21 ± 2°C and humidity of 60 ± 5%. A high-precision analysis balance (Huachang HC311, measuring range 50 g, accuracy 0.1 mg) was placed on a vibration isolation table within a sealed box to prevent inaccurate readings caused by fluctuations in ambient air flow. The platform for experimental verification is shown in Fig 3a and b.

### 2.4. Control algorithm

The precise drug delivery control method should ensure that, after the controller issues the injection command, the minimum accuracy of 0.05 U can be quickly achieved and the mean deviation of the infusion dose is less than 5%. The influence of the elastic deformation of the reservoir and infusion pipeline and the liquid itself on the administration accuracy was ignored, and the influence of the system control strategy on the administration accuracy was emphatically studied. In this study, the insulin delivery control algorithm is used to predict the quasi-dynamic motor inertial propulsion; that is, which is regarded as the inertial propulsion after the current motor braking, monitors the inertial propulsion after the last motor braking, and the prediction is transmitted successively. PID speed control is used to control the insulin infusion dose by precisely controlling the motor speed and running time. In order to solve the problem that the motor exceeds the predetermined angle due to inertia, the LSTM deviation prediction is established. The predicted value of LSTM is evenly allocated to each insulin injection to compensate for the rotational inertia of the motor, thus improving the injection accuracy of the insulin pump. For more information on the insulin delivery algorithm, PID algorithm, and recurrent neural networks, please refer to the supplementary information.

## 3. Results and analysis

**Fig 4:**
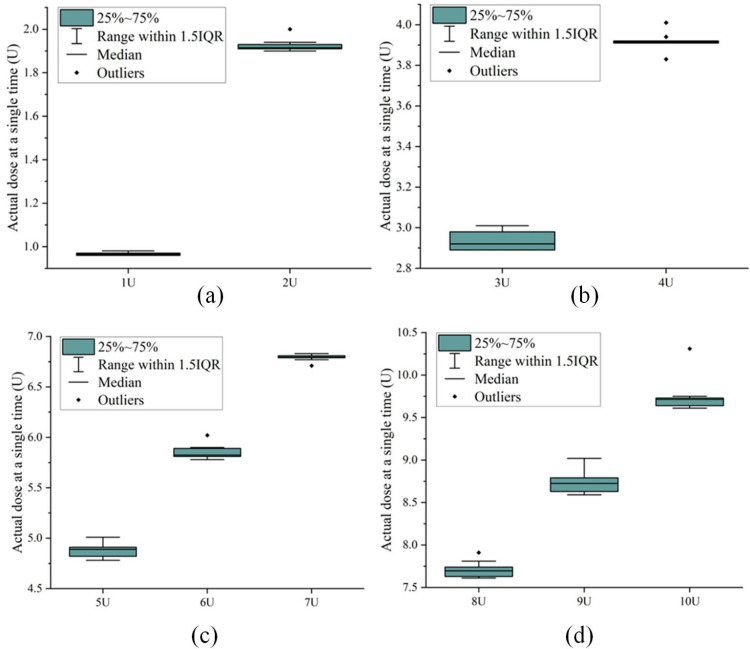
Boxplot of theoretical and actual injection doses. (a) 1U and 2U. (b) 3U and 4U. (c) 5U-7U. (d)8U-10U.

In the actual injection process, mechanical clearance, friction, reservoir characteristics, pipeline elastic deformation, and other factors may cause the theoretical dose and the actual injection dose to be inconsistent inconsistent. The unmodified PH300 insulin pump performs infusion completely under the control of the insulin delivery algorithm. In this experiment, a single large dose was administered, the dose range was 1 ~ 10 U (step size of 1 U), and 10 repeated experiments were performed for each dose, and the results of each experiment were measured 6 times and meant. The maximum and mean infusion deviations were recorded and analyzed for each dose. The experimental data presented in Table 2 reveals that occasional variations in dosage measurement occur due to factors such as viscous forces of the needle on the liquid, evaporation, and measurement deviations. And the boxplot of theoretical and actual injection doses was shown in Fig 4. The observed deviation fluctuates between 3.67% and 4.87%, meeting only the minimum design requirements for the targeted delivery accuracy of the insulin pump (deviation ≤ 5%).

**Table 2:** Performance comparison between theoretical and actual injection doses.

Set	Actual dose at a single time (U)										Maximum deviation	Mean deviation
	No.1	No.2	No.3	No.4	No.5	No.6	No.7	No.8	No.9	No.10		
1 U	0.96	0.96	0.97	0.96	0.97	0.98	0.96	0.96	0.98	0.97	4.00%	3.30%
2 U	1.91	1.91	1.90	1.94	1.92	1.93	1.91	2.00	1.93	1.91	4.50%	3.70%
3 U	2.91	2.89	2.89	3.01	3.01	2.97	2.98	2.89	2.91	2.93	3.67%	2.77%
4 U	3.92	3.91	3.92	3.83	3.91	3.94	3.92	3.91	3.91	4.01	4.25%	4.13%
5 U	4.87	4.78	4.91	4.82	4.78	4.87	4.93	5.01	4.91	4.91	4.40%	2.46%
6 U	5.89	5.78	5.90	5.81	5.79	5.81	5.83	5.88	6.02	5.82	3.67%	2.85%
7 U	6.79	6.71	6.77	6.79	6.80	6.81	6.81	6.83	6.81	6.79	4.14%	2.98%
8 U	7.73	7.61	7.62	7.70	7.63	7.65	7.69	7.91	7.74	7.81	4.87%	3.64%
9 U	8.63	8.61	8.59	8.76	8.73	9.02	8.72	8.67	8.86	8.79	4.56%	3.19%
10 U	9.72	9.61	9.75	10.31	9.73	9.71	9.72	9.64	9.68	9.61	3.90%	3.14%

### 3.1. Pump structure optimization

In this study, the motor control scheme is to add a photoelectric code plate at the end of the motor shaft, and use the frequency of the feedback pulse of the code plate to control the speed of the motor, and precisely control the motor speed and operation time to further the drug delivery accuracy. The structure of the one-stage insulin pump and the two-stage insulin pump is different, because of their maximum stroke, the inside diameter of the medicine reservoir, the gear reduction ratio, and the screw pitch. So, the number of pulses that need to be set for a single infusion of 0.05 U of the two insulin pumps is also different. The main technical parameters of the mechanical structure are shown in S2 Table in S1 File. When the dosage of the two insulin pumps is 0.05 U, the number of pulses that need to be fed back on the code disk is calculated:


$$N=\frac{4\cdot N_{0}\cdot i}{P\cdot \pi \cdot d^{2}}\times 0.5U\;$$ (2)

where *N* represents the number of feedback pulses of the code-disc, *d* represents the internal diameter of the drug-storage-device (mm), *i* represents the reduction ratio of the gear group, *p* represents the screw pitch (mm), and *N*_*0*_ represents the number of lines of the code-disc.

Through calculation, it can be seen that a single infusion of 0.05 U insulin by a one-stage insulin pump requires 10.5 pulses, while a single infusion of 0.05 U insulin by a two-end insulin pump requires 465 pulses. This shows that the two-stage insulin pump can achieve more refined infusion under the same number of code-lines. In addition, under the same infusion dose, the one-stage-screw propulsion distance is shorter. Due to the elastic deformation of the drug storage device and infusion pipeline, the influence of the liquid itself on the delivery accuracy, and the influence of the mechanical transmission gap and other factors, there may be no liquid outflow at the output end of the small-dose infusion. After a large number of small-dose-repeated experiments, it was found that the minimum effective infusion dose of a 1-stage insulin pump was 0.095 U, and the minimum effective infusion dose of a 2-stage insulin pump was 0.047 U. The two-stage insulin pump reduces the minimum effective infusion dose by approximately 50.52%, providing a more refined treatment option for patients with insulin dose sensitivity.

In order to verify the enhanced performance of the improved insulin pump, this study conducted a single small dose and a single large dose infusion test on the improved insulin pump. The experimental parameters were designed as shown in Table 3. Each dose was repeated 10 times, each experiment was measured 6 times, and the mean was taken as the actual result. The maximum single injection deviation, minimum single injection deviation, and mean injection deviation at each dose of primary and secondary pumps were calculated. The single infusion deviation and mean infusion deviation are calculated in the following format:

**Table 3:** Comparison of single infusion accuracy between single-stage and two-stage pumps.

Infusion dose	Maximum deviation		Min deviation		Mean deviation	
	One-stage	Two-stage	One-stage	Two-stage	One-stage	Two-stage
0.1 U	6.17%	6.43%	5.64%	5.51%	5.78%	5.67%
0.2 U	5.54%	5.14%	5.03%	3.68%	5.14%	4.78%
0.3 U	5.74%	5.23%	4.08%	4.01%	4.39%	4.34%
0.4 U	4.78%	5.04%	4.33%	4.16%	4.58%	4.52%
0.5 U	5.14%	4.68%	4.07%	4.15%	4.45%	4.35%
0.6 U	4.81%	4.65%	3.59%	3.26%	3.75%	3.61%
0.7 U	4.96%	4.77%	4.01%	3.04%	4.39%	3.86%
0.8 U	5.03%	4.63%	3.17%	3.05%	3.66%	3.27%
0.9 U	4.52%	4.55%	3.35%	3.32%	3.72%	3.66%
1.0 U	3.77%	3.58%	3.46%	2.89%	3.60%	3.26%


$$Single\;infusion\;deviation=\frac{Theoretical\;dose-Actual\;dose}{\;Theoretical\;dose}\times 100\;\%$$ (3)

$$Mean\;deviation=\frac{Sum\;of\;single\;infusion\;deviations}{Number\;of\;infusions}\times 100\;\%$$ (4)

According to the experimental results, the accuracy of insulin pump infusion before and after improvement was compared. As shown in Table 3, for a single small-dose infusion, the infusion accuracy of the two-stage pump is superior to that of the one-stage pump. At 0.1 U, 0.2 U, 0.3 U, 0.4 U and 0.5 U, the mean infusion deviation of the two-stage insulin pumps was reduced by 2.60%, 5.87%, 2.65%, 3.99% and 2.77%, respectively. Taking 0.1U as an example, the average deviation reduction rate is calculated as: (5.77%−5.62%)/5.77%.

In addition, in order to verify the improvement amplitude and significance of the injection accuracy of the two-stage pump compared with the one-stage pump, a paired T-test was carried out on the average error of the one-stage and two-stage pumps in Table 3 to evaluate whether the difference between the mean deviation is significant. In order to simplify the data complexity of the testing process, T-tests were carried out for all the mean deviations expanded by a factor of 100. T-test is a commonly used statistical test, mainly applied to small samples (n < 30), the population standard deviation σ is unknown, and the data approximate a normal distribution. Its main purpose is to infer whether the difference between two averages is significant by hypothesis testing [51,52]. The statistical structure and test results are shown in S3 and S4 Tables in S1 File. It can be seen from the test results in S4 Table in S1 File that in the paired test of 10 groups from 0.1 U to 1.0 U, the differences in 8 groups were significant (P-value < 0.05). The comparison group with a statistical difference accounted for 80%. Taking the paired test under 0.2 U as an example, the statistical sample size of paired samples was 10 pairs, the mean deviation of infusion accuracy of one-stage and two-stage pumps was 5.14 and 4.78, the standard deviation was 0.14937 and 0.40368, and the standard error mean was 0.04723 and 0.12765, respectively. For the paired sample test, the mean difference of the mean deviation of one-stage and two-stage pump infusion is 0.35300, the standard deviation is 0.35923, and the standard error mean is 0.11360. The 95% confidence interval of the difference is [0.09602, 0.60998], t = 3.107, degrees of freedom (DOF)=9, P = 0.013. The difference in the average infusion deviation of the pump before and after the improvement is statistically significant. It can be considered that the improvement of the mechanical structure has a significant effect on reducing the infusion deviation of the insulin pump.

In addition, the accuracy of low basal rate infusion is a critical design parameter for insulin pumps. This paper conducted comparative experiments to assess basal rate infusion accuracy, as detailed in Table 4. Among insulin pump users, the average base rate is between 0.4-0.9U h-1 [53,54]. However, due to limitations in experimental equipment, this study could only measure accuracy at a constant reference rate of 0.5 U h-1. The base rate experiment ran continuously at 0.5 U h-1 for 24 hours, with the balance reading recorded every 30 minutes. To enhance data readability, time measurement nodes were established, as indicated in Table 4. The accuracy of low basal rates infusion for both one-stage and two-stage pumps is presented in Table 4, encompassing a total of 14-time nodes. In addition, under constant low basal rate infusion, the infusion accuracy of the two-stage pump is significantly better than that of the one-stage pump. After 6h, 12h, 18h and 24h of low basal rate infusion, the overall infusion deviation of the two-stage pump was reduced by 15.24%, 7.33%, 17.34%, and 12.85%, respectively.

**Table 4:** Accuracy comparison of single-stage and two-stage pumps for low base rate infusion.

Times	Cumulative dose		Total deviation	
	One-stage	Two-stage	One-stage	Two-stage
0.5h	0.23 U	0.23 U	8.00%	8.00%
1.0 h	0.47 U	0.47 U	6.00%	6.00%
2.0 h	0.94 U	0.96 U	6.00%	4.00%
4.0 h	1.89 U	1.92 U	5.50%	4.00%
6.0 h	2.87 U	2.89 U	4.33%	3.67%
8.0 h	3.78U	3.81 U	5.50%	4.75%
10 h	4.76 U	4.79 U	4.80%	4.20%
12 h	5.73 U	5.75 U	4.50%	4.17%
14 h	6.73 U	6.78 U	3.86%	3.14%
16 h	7.67 U	7.72 U	4.13%	3.50%
18 h	8.60U	8.67 U	4.44%	3.67%
20 h	9.54 U	9.63 U	4.60%	3.70%
22 h	10.61 U	10.71 U	3.55%	2.64%
24 h	11.57 U	11.62 U	3.58%	3.12%

### 3.2. BP-PID motor speed simulation

For insulin pumps, if the motor is not controlled by external factors, it will affect the delivery accuracy of the system. In order to control the above factors, a photoelectric encoder is introduced into the output shaft of the motor, and a speed detection loop is formed by using the pulse frequency fed back by the photoelectric encoder. By controlling the steady speed and running time of the motor, the infusion accuracy of the insulin pump is controlled. System controller parameters are designed, and a feasible motor control strategy is established by simulation to ensure the accuracy of drug delivery. The transfer function of motor speed and input voltage is approximated as a first-order inertial link. The system velocity loop is designed as a typical second-order system.

**Fig 5:**
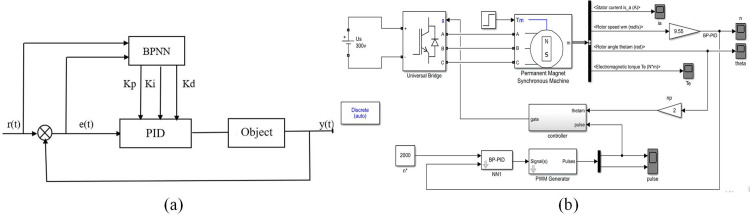
The design of the brushless direct current motor (BLDCM) closed-loop speed regulating system. (a) BP neural network combined with PID controller structure. (b) BLDCM closed-loop single-speed system is controlled based on BP-PID.

In the design of the BLDCM closed-loop speed regulating system, a PID controller is adopted in this study. However, in order to achieve higher performance and adaptability, this simulation introduces the BP neural network algorithm, which is closely integrated with the PID controller (BP-PID), so as to realize the adaptive adjustment of PID parameters. The structure principle of BP-PID is shown in Fig 5a. First of all, the simulation is based on the first-order system to carry out experimental verification and evaluate the performance of the PID controller based on the BP neural network. The experimental results show that the controller is stable and efficient. After validation, the strategy is migrated to the brushless DC motor speed control system. Finally, the BLDCM speed single closed-loop system based on BP-PID control was successfully constructed, as shown in Fig 5b.

**Fig 6:**
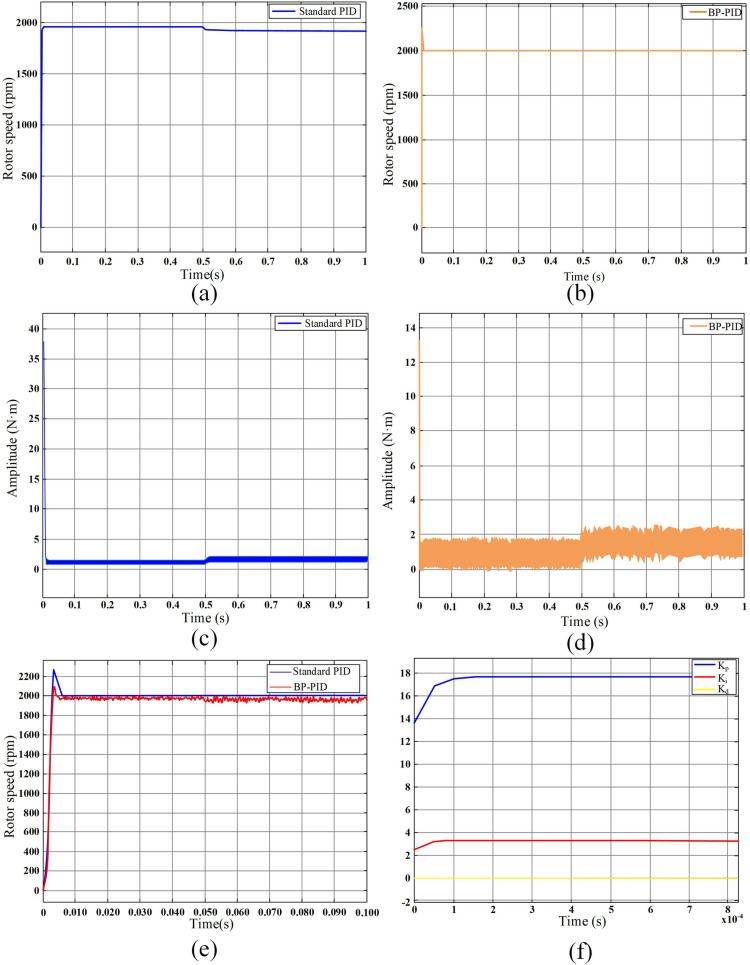
Simulation performance and control effect of BP-PID. (a) motor speed waveform with standard PID. (b) motor speed waveform with BP-PID. (c) electromagnetic torque waveform with standard PID. (d) electromagnetic torque waveform with BP-PID. (e) comparison of motor speed overshoot between standard PID and BP-PID. **(f)** PID adaptive parameter waveform diagram.

During the use of the insulin pump, the load on the motor will also change with the change in pressure and dosage in the reservoir, which will affect the accuracy and smoothness of the infusion. Therefore, it is essential to ensure that the motor can operate stably under different loads and meet performance requirements. In the BLDCM simulation speed regulation system, a 1 N·m load is first applied to the motor, and then the load is suddenly increased to 1.5 N·m at 0.5 seconds. Its speed and electromagnetic torque waveform are shown in Fig 6.

As can be seen from Fig 6, the closed-loop speed-regulation system using a BP-PID controller and the closed-loop speed-regulation system with a standard PID controller have similar change trends in speed, A-phase current and motor torque. To more clearly compare the speed changes of the two, the simulation time was changed to 0.1 s and the load mutation time to 0.005 s for comparison, and the result was shown in Fig 6. As can be seen from Fig 6e, when the load is 1 N·m, the overshoot of the BP-PID controller is much smaller than that of the traditional PID controller, and the stable state is reached quickly. In the case of sudden load changes, the former is shorter than the latter adjustment time, and the anti-interference ability is better. In Fig 6f, the blue line represents the proportional parameter K_p_, the orange line represents the integral parameter K_i_, and the green represents the differential parameter K_d_. The proportional and integral parameters are automatically adjusted to adapt to the BLDCM closed-loop speed-regulation system under the adjustment of the BP neural network algorithm, and the differential parameter is set to 0.

### 3.3. LSTM prediction compensation

**Fig 7:**
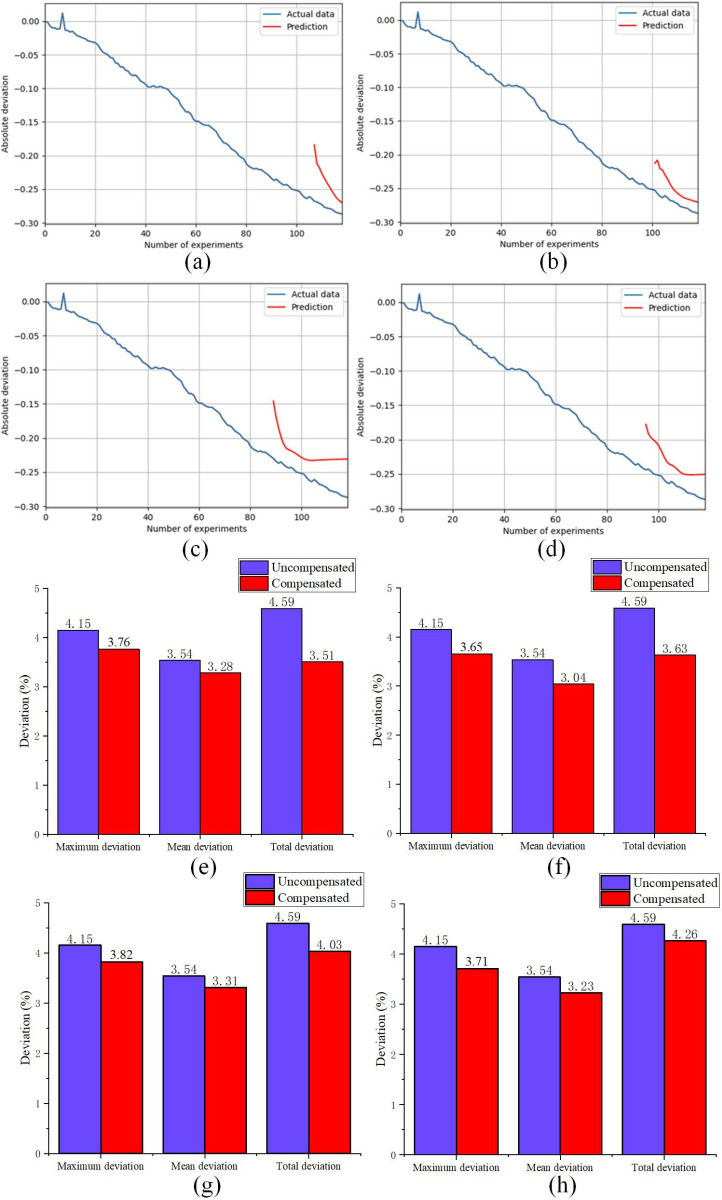
Prediction (a-d) and infusion deviation (e-f) results under different training sets and test sets. (a) and (e) training set 90%, test set 10%. (b) and (f) training set 85%, test set 15%. (c) and (g) training set 80%, test set 20%. (d) and (h) training set 75%, test set 25%.

In order to solve the problem that the motor exceeds the predetermined angle due to inertia, the LSTM deviation prediction model is established. The experimental deviation of each infusion is input into the LSTM prediction model, and then the predicted value of LSTM is evenly distributed to each injection of insulin to compensate for the deviation caused by the moment of inertia of the motor, thereby improving the injection accuracy of the insulin pump. Deviations of 120 experimental data (each 2U infusion) were input into the LSTM model, and different coefficient divisions of the training set and test set were used for prediction. The experimental deviation accumulation and LSTM prediction deviation results obtained are shown in Fig 7.

As can be seen from Fig 7, the predicted value is close to the actual value, and the predicted result is better. The accumulated deviation prediction value of the 120th infusion experiment was evenly distributed to compensate for the verification test in these 120 tests, so as to explore the division of the training set and the test set in the optimal case. As can be seen from Fig 7, the mean compensation to each prediction under the division of different training sets is 0.221 U, 0.226 U, 0.208 U, and 0.198 U, respectively. The compensation value is compensated in each test, and the test result is shown in Fig 7. Each subgraph shows, from left to right, the single maximum deviation, the mean deviation of 120 infusions, and the total deviation of 120 infusions. The experimental deviations after compensation are all smaller than those without compensation, and among the four training set divisions, the maximum infusion deviation under the 85% training set is the smallest, which is 3.65%; the mean infusion deviation under the 85% training set is the smallest, which is 3.04%; and the overall infusion deviation under the 80% training set is the smallest, which is 3.85%. Because insulin infusion is a long-term, frequent process, the mean infusion deviation is more important to the effect of insulin infusion. Therefore, setting the training set to 85% and the test set to 15% is the most appropriate parameter choice for the insulin infusion deviation prediction model.

In the training of the LSTM model, in addition to selecting the appropriate training set and test set, it is also necessary to set parameters such as the number of iterations and the number of hidden nodes, which will significantly affect the learning effect of the neural network. The structure of the network model is composed of an input layer, a hidden layer, and an output layer. The hidden layer is used for linear segmentation of various data. Theoretically, the increase of the number of hidden layers will improve the calculation accuracy. But in reality, the more layers you have, the more meaningless parameters you have. The model can be divided into a dense layer structure and an LSTM layer structure. The goal of the dense layer is to make nonlinear changes to previously extracted features. On this basis, the correlation between the features is extracted and mapped to the output space. Therefore, when the default iteration number is 300 and the batch number is 64, L represents the LSTM layer and D represents the Dense layer, and the influence of different hidden layer structures on the prediction accuracy of the model is discussed. S5 Table in S1 File shows the tuning process. It can be clearly seen that the effect of 3-layer model structure is much better than that of 4-layer model. The model structure has achieved a good effect in the three-layer structure, but increasing the number of model layers has little impact on the model accuracy, but the running speed is greatly reduced, so this work chooses the three-layer network structure. At the same time, among the three-layer-model-structure, LDL-model has the best result, with a mean absolute percentage error (MAPE) of 20.25% and a root mean square error (RMSE) of 59.8416. Therefore, it is used as the network structure of the LSTM model.

After the model structure is determined, the optimal prediction effect of the LSTM model is obtained by optimizing the number of iterations, the number of batches and the number of hidden layer neurons of the network model. For example, the choice of epoch (training times) needs to balance overfitting and underfitting. If there are too few epochs, the model may not be able to learn the data features adequately; too many may lead to overfitting. In addition, the effect of dataset size on the hyperparameters also needs to be considered. If the dataset is larger, larger batches and more hidden units may be more appropriate, and vice versa may need to be adjusted. Therefore, the optimal parameter combination is performed by using the grid search method, as shown in Table 5. As can be seen from Table 5, the LSTM model achieves the optimal combination, when the number of training times is 300, the number of batch Processing is 256, and the number of hidden-layer neurons is 32. Under this hyperparameter condition, the LSTM model achieved minimum values of MAPE and RMSE, which are 23.09 and 75.1967, respectively.

**Table 5:** LSTM parameter combination by using the grid search method.

Training times	Batch quantity	Number of hidden layer neurons	MAPE	RMSE
100	128	32	25.36	67.8240
100	128	64	28.57	77.2327
100	256	32	28.83	79.8019
100	256	64	30.04	75.6231
300	128	32	32.23	97.8158
300	128	64	28.87	77.7063
300	256	32	23.09	75.1967
300	256	64	26.98	74.4770
500	128	32	24.10	75.2343
500	128	64	28.36	83.2820
500	256	32	26.05	75.7170
500	256	64	29.11	82.5310
700	128	32	24.72	75.6343
700	128	64	25.17	75.6841
700	256	32	27.53	76.4102
700	256	64	26.78	76.0348

**Fig 8:**
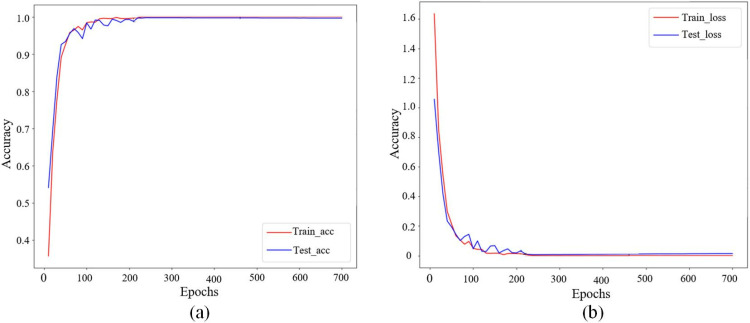
Accuracy and loss of LSTM model training and testing. (a) LSTM training and testing accuracy. (b) LSTM training and testing losses.

Set the number of iterations of the LSTM model to 700. In the training process, the test set is used for one test each iteration, and the accuracy and loss of the model on the training set and test set are recorded. The accuracy rate is the mean proportion of the data samples correctly predicted in each iteration in the total samples, and the loss value is the mean value of the predicted value and the actual value after the cross-entropy function calculation. The record is plotted as a learning curve after 700 iterations. The training and test accuracy of the LSTM model is shown in Fig 8a, and the training and testing loss is shown in Fig 8b. When the training reaches about 100 times, the accuracy rate begins to converge and gradually becomes flat. At the 100th to 200th iteration, the curve fluctuates continuously in a small range, which indicates that the model is not optimal. When the number of iterations reached 220, the training curve gradually stabilized. At the same time, the training loss in the loss curve in Fig 8b drops to 0.00006 and is basically unchanged. When the iteration reaches 280 times, the test accuracy of the model also gradually stabilizes, and the accuracy of the model finally reaches 97.53% on the training set. Therefore, adjusting the training number to 300 times can reduce the training time.

## 4. Discussions

### 4.1. Improvement of mechanical structure

Turning a single-stage driving screw of the insulin pump into a dual-stage design represents a forward-looking and innovative engineering improvement. It significantly enhances the precision of insulin delivery while reducing the minimum infusion dosage of the insulin pump, thereby offering a more finely tailored treatment approach for diabetes patients. The dual-stage screw drive system boasts higher control precision, enabling insulin to be administered with smaller dosage increments. This is particularly crucial for patients who require fine-tuning of their insulin doses, especially children and elderly individuals. In contrast, the traditional single-stage screw drive system may incur deviations and inaccuracies during low-dosage infusions, a risk effectively mitigated by the dual-stage system’s reduction in dosage increments. In addition, the optimization of the screw’s anti-backlash structure in the two-stage screw drive system effectively minimizes the occurrence of screw dead zones or jamming, thereby enhancing both the precision of insulin delivery and the safety of its use. This improvement reduces potential medical risks.

The enhancement of the insulin pump with a dual-stage screw drive, as explored in this study, not only improves delivery precision but also lowers the minimum infusion dosage. It provides diabetes patients with a more finely tailored treatment approach. This innovation holds broad application prospects in improving the field of diabetes management and yields numerous positive impacts. The dual-stage screw drive enables more precise control over drug release and infusion rates, decreasing the likelihood of infusion deviations. This can enhance diabetes patients’ blood glucose control and reduce the risk of high and low blood sugar events. Furthermore, due to the increased precision of the insulin pump, healthcare professionals can make finer adjustments to the pump’s parameters to meet the unique needs of each patient. This opens up opportunities for personalized treatment, adapting insulin infusion to patients’ lifestyles and blood sugar fluctuations. By reducing infusion deviations and better managing blood sugar levels, diabetes patients can experience an improved quality of life, with fewer discomforts and complications associated with diabetes. However, it is worth noting that this enhancement requires precise engineering design and good precision machining capabilities to ensure the stable operation of the new mechanical structure under a variety of conditions [55,56]. Additionally, subsequent clinical trials and patient feedback will be crucial steps in determining the actual effectiveness and feasibility of this improvement.

As a medical device that mimics the body’s natural insulin secretion, the design goal of insulin pumps is to restore the insulin secretion pattern in the human body as much as possible, so as to achieve effective control of blood glucose levels in diabetic patients. Low basal rate infusion refers to the insulin pump’s demand to continuously infuse insulin at a low and stable rate when simulating insulin secretion in the non-fed state of the human body, which is also a very important function. Due to the limitations of the experimental equipment, this study could only measure the accuracy at a constant reference rate of 1.0 U/h. In practice, insulin pumps may need to be infused at different basal rates, so further studies should consider assessing the infusion accuracy of insulin pumps at different basal rates. In addition, in the experiments of this investigation, we replaced insulin with distilled water, which may lead to inaccurate calculation of the pump delivery volume, and thus affect the accuracy of dose control. In order to verify the feasibility of replacing insulin with distilled water, we firstly did the experiment, and there was no significant difference between the two from the statistical data.

### 4.2. BLDCM speed simulation performance

One of the most central approaches to diabetes treatment is extracorporeal insulin infusion. Improvements in insulin infusion accuracy optimize glycemic control through the following mechanisms. First, precise dose output (error <±5%) avoids dose bias caused by scale misreading or uneven pushing of conventional syringes and reduces alternating fluctuations of hyperglycemia and hypoglycemia. Second, dynamic and precise infusion can match the complex metabolic demands: mealtime insulin improves the absorption efficiency through high-dose injection, so that the postprandial glucose peak is lowered; the basal rate is precisely regulated to maintain the standard deviation of the 24-hour glucose curve within a reasonable range. Finally, with regular testing of blood glucose, diabetic complications can be effectively delayed. Thus, the improvement of injection precision systematically enhances the steady-state control of blood glucose through the three-dimensional optimization of dosage, timing, and dynamic response. This means that insulin injection accuracy is essential for glycemic control.

This study proposes a novel control strategy for addressing the infusion issues of a piston-type insulin pump by combining neural networks with traditional PID controllers. This combination effectively leverages the nonlinear modeling and learning capabilities of neural networks, as well as the stability and real-time performance of PID controllers [57], to effectively balance the trade-off between motor speed smoothness and infusion precision. Infusion precision is of paramount importance for diabetes patients. It is not only necessary to ensure that insulin is delivered accurately according to the prescribed dose, but also to avoid over-infusion or under-infusion, to prevent the occurrence of hypoglycemia or hyperglycemia events. Therefore, this research has profound implications for improving the feasibility and effectiveness of insulin therapy.

The infusion precision of a piston-type insulin pump primarily depends on the precise control of the rotation of the BLDCM. Currently, accurate insulin infusion is generally achieved by controlling the stable speed and operating time of the BLDCM. As the insulin pump requires frequent start-up and braking, ensuring the smoothness of BLDCM speed is crucial to guarantee insulin precision. This study combines a BP neural network with a traditional PID controller to form a BP-PID controller, which adjusts the PID’s proportional, integral, and derivative parameters online through the BP neural network. The control performance of open-loop speed control, closed-loop speed control, and BP-PID-based closed-loop speed control of the BLDCM is compared, with a primary focus on the critical parameter of motor speed for an in-depth understanding of the effectiveness of various control strategies. Simulation results indicate that the closed-loop system outperforms the open-loop system significantly. The closed-loop system, through real-time feedback mechanisms, can more accurately control the motor speed, thereby reducing system deviations and improving stability. Particularly, when facing external disturbances and varying loads, the closed-loop system can respond more rapidly, effectively suppressing interference, and maintaining a stable operational state. Additionally, BP-PID control offers unique advantages, not only achieving precise control but also exhibiting smaller overshoot and faster response times. Inevitably, BP-PID control also has some shortcomings. For example, BP-PID exists modeling dependence, while standard PID does not require an accurate model of the system, and the tuning parameter is simple. BP-PID requires pre-training a neural network, which relies on a large amount of historical data, and the initial modeling cost is high. In addition, BP-PID also has real-time limitations. The online parameter optimization of BP-PID increases the computational load, which may trigger a millisecond delay in low-end microcontroller units (MCUs). Whereas standard PID has a constant computational load and is suitable for high real-time scenarios. Thus, it needs to continue to be improved or overcome in future research.

### 4.3. LSTM prediction for semi-closed loop pumps

In order to address the issue of the motor exceeding the predetermined angle due to inertia, an LSTM deviation prediction model has been established. By analyzing the available data, we found that LSTM is more suitable to handle this task through multilayer gating structure and nonlinear activation functions compared to linear statistical models (e.g., exponential smoothing) and shallow machine learning methods (SVM). In this study, the deviation prediction outputs of the LSTM model are evenly distributed to each insulin injection to compensate for the motor’s rotational inertia. It means that with every insulin injection, the predictions from the LSTM model are considered to reduce the precision deviation of the insulin pump. Experimental validation results demonstrate that the LSTM model performs well in predicting insulin pump infusion deviations. It should be noted that the optimal number of LSTM layers is not the more the better. When determining the number of LSTM layers, experiments show that the three-layer structure achieves an optimal balance in the injection prediction task, which may be attributed to the following two reasons. Firstly, when the number of layers increases from 3 to 4, too deep a network leads to gradient attenuation, and there may be saturation in the model performance, resulting in the deep network indistinctively improving the feature extraction capability. Two, the network with 4 layers may be at risk of overfitting, and we have also revealed in experiment that the four-layer model has fluctuating variance in the prediction of the test set. In the management of insulin pump infusion, the combination of the LSTM model with the deviation allocation method provides an effective approach to enhancing accuracy and reliability. This approach holds the potential to assist diabetes patients in better managing their blood glucose levels and reducing the risk of infusion deviations. However, its stability and safety need further validation in real-world applications.

The core concept of a semi-closed-loop insulin pump is to maintain patient autonomy while utilizing real-time blood-glucose monitoring data to provide more accurate insulin delivery control [58,59]. A semi-closed-loop insulin pump combines elements of open-loop and closed-loop control. It allows patients to set basic parameters for insulin delivery, such as basal rates and post-meal bolus amounts, but can also automatically adjust them based on real-time blood-glucose measurements in specific situations. In the future, the use of LSTM for deviation prediction compensation during insulin infusion, particularly at critical infusion points, holds promise for improving insulin delivery accuracy. However, the challenge of how to automatically collect past infusion data for deviation calculation in an offline setting and input it into the LSTM model database for training and prediction is a topic that this research aims to address. It is worth noting that LSTM algorithms also have drawbacks. LSTM requires a large amount of labeled data for training, in which the data acquisition cost is high and there are individual differences, and it has interpretability defects compared to SVM and so on. In addition, deploying LSTM models in low-power embedded systems faces the challenges of arithmetic adaptation, power optimization and real-time guarantee. In the future, we will adopt model compression techniques (e.g., 8-bit integer quantization + weight pruning), which can reduce the number of LSTM parameters. In this way, computation can be reduced, power consumption can be optimized, and the real-time requirement can also be satisfied.

## 5. Conclusions

In this study, the injection accuracy of the commercially available PH300 insulin pump was enhanced by improving the mechanical structure of the insulin pump and adopting the BP-PID controller and LSTM prediction deviation compensation. The following conclusions were obtained through comparative experiments.

(1) It could be seen from the experimental results that the minimum effective single injection dose of the improved PH300 was 0.047 U, which was reduced by 50.52% and could provide more accurate treatment options for insulin dose-sensitive patients. The low dose (0.1U-0.9U) mean infusion deviation was reduced by 4.91%, and the overall infusion deviation was reduced by 12.85% after the low basal rates and 24h window.
(2) By comparing the BLDCM single-speed closed-loop simulation performance of the BP-PID controller with that of the traditional PID controller, it was found that the BP-PID controller had less overshoot, faster stability and shorter regulation time when the 1 N·m load suddenly changed to 1.5 N·m. The optimal adaptive PID parameters K_p_, K_i_ and K_d_ were 17.8, 3.6, and 0, respectively. The proposed BP-PID controller showed significant performance advantages in insulin pump applications, especially in handling load variations and improving injection accuracy. In the future, BP-PID control will be integrated into a new generation of insulin pump products.
(3) Compensating for the LSTM-predicted deviations effectively improved the precision of insulin pump infusion. When LSTM adopted the LDL-model structure, the best predictive performance was achieved with 300 training epochs, a batch size of 256, and 32 hidden-layer neurons, with an 85% training-dataset split and a 15% testing-dataset split. The maximum infusion deviation, mean infusion deviation, and total infusion deviation were 3.65%, 3.04%, and 3.63%, respectively.

## Supporting information

S1 File Algorithm design, pump mechanical structure parameters and test results. (DOCX)

## Abbreviations

PID: Proportional-integral-derivative control
BP-PID: PID controller based on Back Propagation neural network
T1D: Type 1 diabetes
LSTM: Long Short-Term Memory
RNN: Recurrent neural network
DC: Direct current
MCU: Microcontroller Unit
DOF: Degrees of freedom
BLDCM: Brushless direct current motor
MAPE: Mean absolute percentage error
RMSE: Root mean square error
SVM: Shallow machine learning methods.
