# Supplementary material for: Prediction and accuracy improvement of insulin pump in-fusion deviation based on LSTM and PID
Source: PLoS One. 2025 Jun 4;20(6):e0324261. doi: 10.1371/journal.pone.0324261 (PMC12136315; doi:10.1371/journal.pone.0324261)
Supplement: S1 File — (DOCX) [file pone.0324261.s001.docx]

*Supporting information*

*1. PH 300 insulin pump main technical parameters*

**Table S1**. PH 300 insulin pump main technical parameters.

| **Type** | **Parameters** |
| --- | --- |
| CPU | Dual-CPU mutual check |
| Propulsion structure | Two stage ram |
| Basal infusion | 0.6~35.0 U h-1 |
| Charging method | Screw automatic reset |
| Infusion method | Four type |
| Memory function | Time and basic settings are not lost |

2. Insulin delivery algorithm

The injection of insulin is completed by the MCU, which drives the motor to push the syringe piston. However, when the MCU sends the brake instruction, the syringe piston will still advance a little due to motor inertia, thus affecting the infusion accuracy. In addition, when the load (pressure in the syringe, drug storage) changes, the amount of propulsion caused by inertia also changes. In this study, the quasi-dynamic prediction of motor inertia can offset the infusion deviation caused by motor inertia to a certain extent. The program flow chart of the quasi-dynamic motor inertial propulsion prediction algorithm is shown in Figure S1.


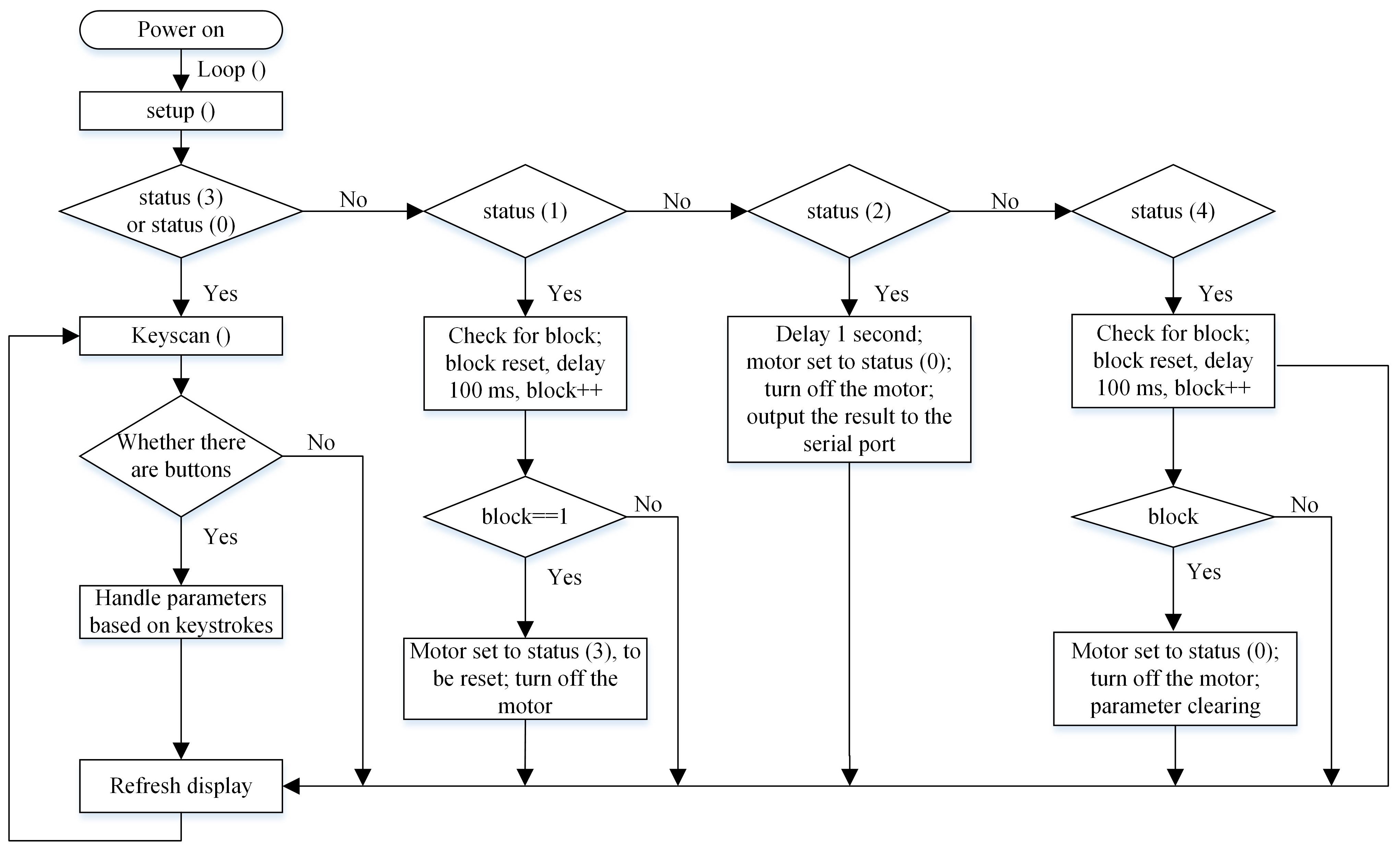


**Figure S1**. The program flow of quasi-dynamic motor inertial propulsion prediction algorithm.

3. PID algorithm

However, for the above algorithm, when the load changes or the system is disturbed, the system output will be difficult to accurately reproduce the input; that is, the motor speed is difficult to maintain at a predetermined value, and the infusion accuracy cannot be guaranteed. In this paper, based on the prediction algorithm of motor inertia, the proportional integral-derivative (PID) algorithm is used to control the motor speed when the motor is started. For the insulin pump system, because the actual operation does not require high speed, the base speed below the adjustment can meet the requirements. In this study, DC pulse width speed (PWM) regulation is adopted to regulate the motor speed. The principle is that a fixed DC voltage changes the voltage on the armature through the control of a switch k with a certain frequency [1], as shown in Figure S2(a). The mean voltage at both ends of the motor armature is shown in equation S1.

$\begin{aligned} U_{d}=\frac{\tau}{T}=\delta_{T}U\#\left( S1 \right) \end{aligned}$

where *T* represents the opening and closing periods, and the closing time of each time is *τ*; *δ_T_* represents duty cycle. As long as the on-off period *T* of the switching device is kept constant and only the pulse width is changed, the mean voltage applied to both ends of the motor armature is also changed. As shown in Figure 4(b), the speed of the motor is proportional to the voltage at both ends of the motor, and the voltage at both ends of the motor is proportional to the duty cycle of the control waveform, so the speed of the motor is proportional to the duty cycle. The larger the duty cycle, the faster the motor rotates, and the maximum motor speed is achieved when the duty cycle *δ_T_* = 1. Therefore, adjusting the pulse width changes the mean armature voltage, which also changes the speed of the motor.


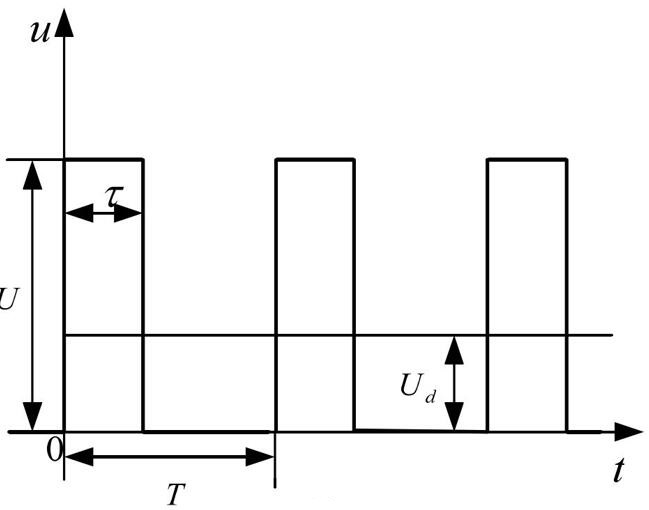

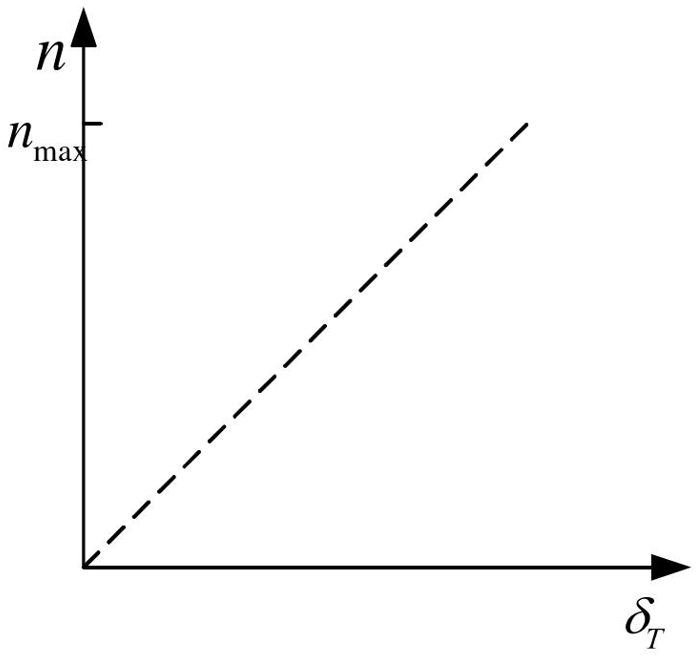


(a) (b)

**Figure S2**. Pulse voltage modulation waveform and speed-duty ratio relation curve; (a) relation curve of opening and closing time and voltage; (b) relation curve between duty cycle and speed.

A PID controller is a general control loop feedback mechanism widely used in industrial control systems. The PID controller corrects the deviation between the measured process variable and the desired set point by calculating and then outputting a corrective action that can adjust the process accordingly [2,3]. Therefore, by integrating the PID controller into the DC motor, the deviation generated by the motor can be corrected, and the speed of the motor can be controlled to the desired speed. The formula is shown in equation (S2) [4].

$\begin{aligned} u\left( t \right)=K_{p}e\left( t \right)+K_{i}\int_{0}^{t} e\left( \tau\right)d\tau+K_{d}\frac{de\left( t \right)}{dt}\#\left( S2 \right) \end{aligned}$

where *e(t)* is the speed difference between the set speed and the output actual speed; *u(t)* is the PWM signal of the DC motor. *K_p_*, *K_i_*, and *K_d_* are proportional coefficients, integral coefficients, and differential coefficients, respectively.

Figure S3 shows the PID controller design of this control system. By calculating the PID equation, the control signal of a DC motor is obtained. In the PID control process, the Arduino microcontroller executes the control signal and implements the instruction code. In this code, the proportional gain (*K_p_*) checks the magnitude of the deviation and responds proportionally. Although there is a large deviation, the size of the motor position will receive a larger response. Integral gain (*K_i_*) is dedicated to reducing steady-state deviations. Derivative gain (*K_d_*) attempts to see the rate of change of the deviation signal. Differential control will reduce the amount of overshoot and make the motor speed of the system response curve change at a faster rate. Finally, the PWM signal is calculated according to the signal change of the DC motor, and the PID gain value is used to improve the system's performance.


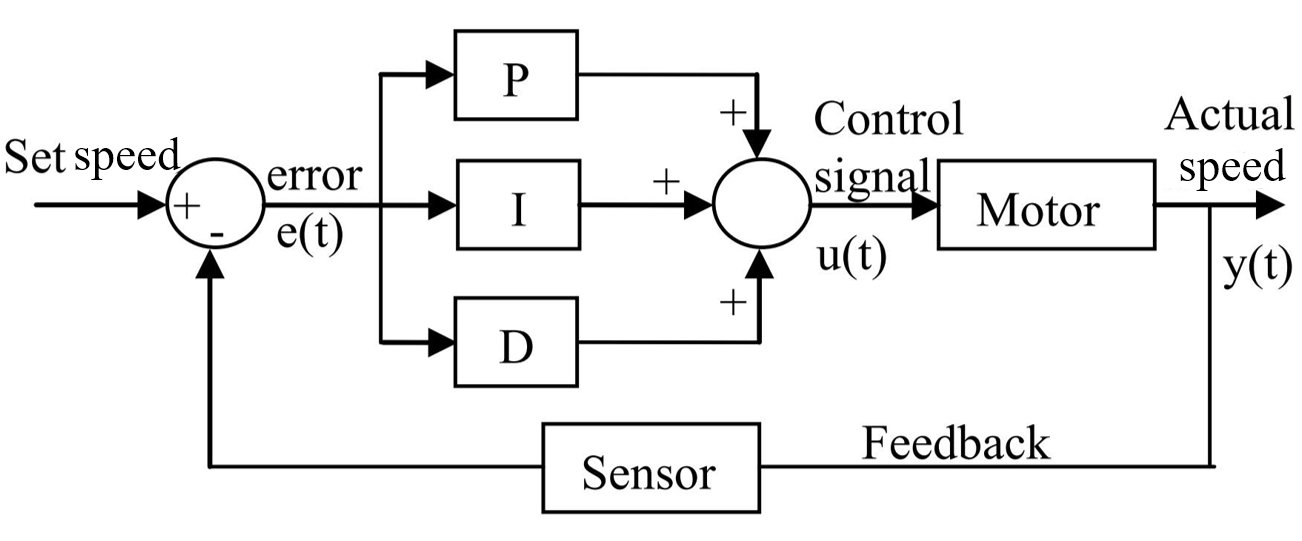


**Figure S3**. Speed control of the DC motor using PID controller.

4. Recurrent neural networks

RNN is one of the most mainstream feature extractors for processing sequence data. It can naturally collect input information from the front to the back in a linear sequence structure and excavate the internal relationships of sequences [5,6]. Figure S4 shows the chronological expansion form of the RNN structure, where x is the input value, y is the output value; h is the state of the hidden layer, U is the weight matrix from the input layer to the hidden layer, V is the weight matrix from the hidden layer to the output layer, and W is the weight matrix from the hidden layer to its next time. The loop layer where the weight W is located is used to connect hidden layers in adjacent time steps and is the key for the recurrent neural network to store memory and transfer state. The main feature of RNN is weight sharing; that is, the value of W in Figure S4 is the same, and the values of U and V are the same. The output result of RNN is not only affected by the current input but also by the previous input, and the specific time interval for transmitting information is determined by the delay length [7].


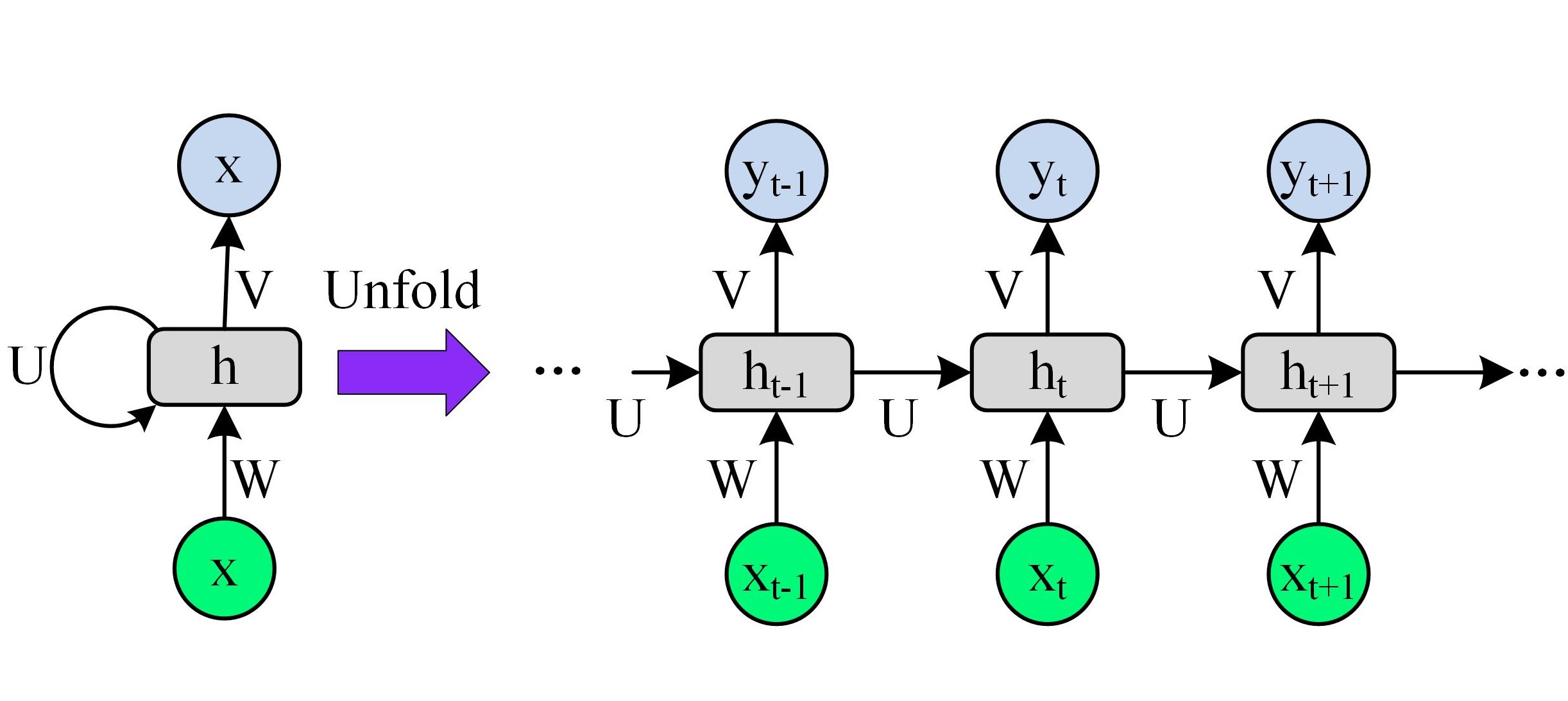


**Figure S4**. RNN typical network structure diagram.

The specific calculation process of RNN can be expressed as:

$$\begin{aligned} y_{t}=g\left( vh_{t}+b_{0} \right)\#\left( S3 \right) \end{aligned}$$

$$\begin{aligned} h_{t}=f\left( ux_{t}+wh_{t-1}+b_{h} \right)\#\left( S4 \right) \end{aligned}$$

where *g (x) and f (x)* are activation functions, and commonly used activation, functions include tansig, relu, tanh, and purelin. 𝑉 is the weight matrix of the output layer. 𝑈 is the weight matrix for input X. 𝑊 is the value of the previous ℎ_𝑡−1_ as the weight matrix for this input.


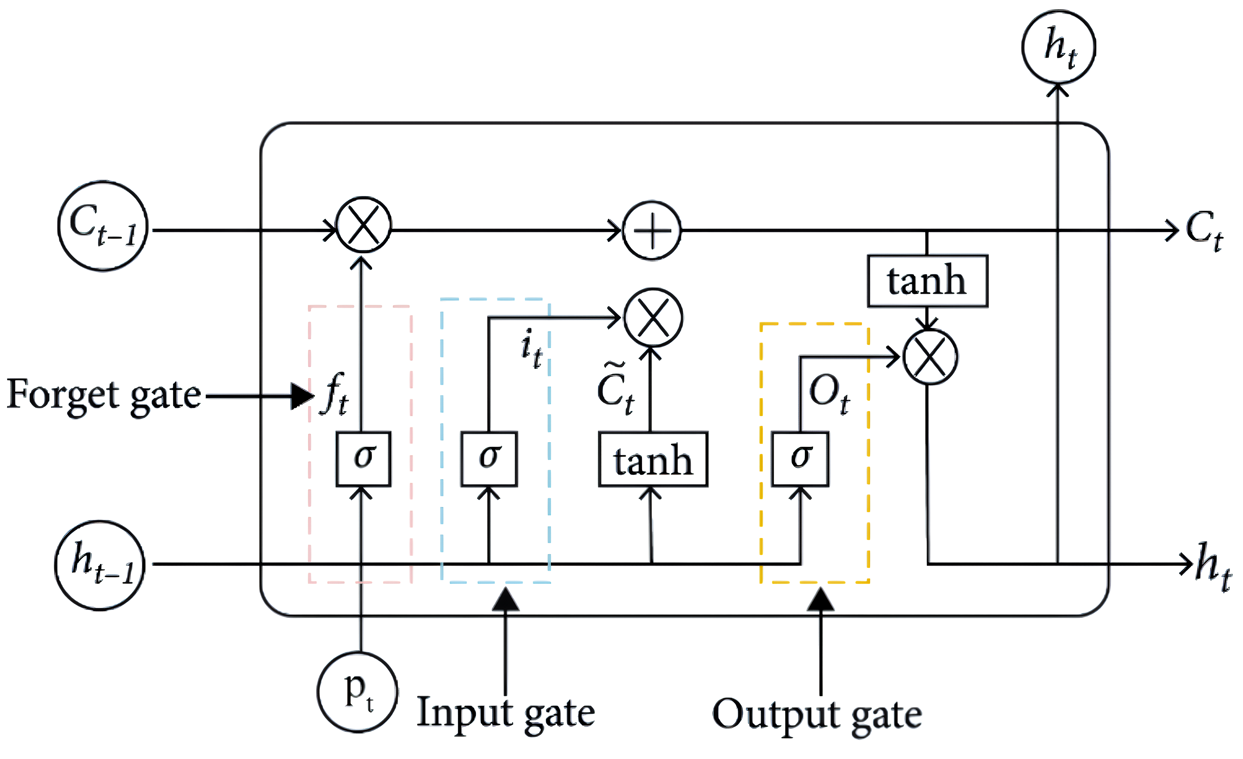


**Figure S5**. LSTM network structure diagram.

Although RNN can solve the whole sequence information, the more recent sequence information has a greater impact on it, and the more distant sequence information has a lower reference value. Therefore, the long-term memory ability of RNN is insufficient, and there may be phenomena such as gradient disappearance and gradient explosion. The LSTM network is improved on the topology of RNN [8]. As a variant of RNN, the LSTM model effectively solves the problem of gradient disappearance caused by the increase of network layers and the passage of time by introducing a controllable self-loop, which is especially suitable for processing tasks with a long delay and time interval [9,10]. Compared to traditional RNN cells, LSTM cells have a more complex structure, as shown in Figure S5.

The LSTM unit changes the cell state information through the hidden layer by introducing a "gate" mechanism, thus realizing the long-term memory of information. Each LSTM unit contains an input gate, an output gate and a forget gate to control information collection and transmission between hidden units [11]. The input gate determines what cell state information will be updated, the forget gate determines what information will be retained, and the output gate can selectively output information as needed. LSTM adds a new unit state 𝐶𝑡 to store the long-term dependent feature state, and relies on three gate control units: the forgetting gate 𝑓𝑡, the input gate 𝑖𝑡 and the output gate 𝑂𝑡 to control 𝐶𝑡 update delivery. The detailed calculation process is given below [12];

**Forget gate**

$$\begin{aligned} f_{t}=\sigma\left( W_{f}\cdot\left[ h_{t-1},x_{t} \right]+b_{f} \right)\#\left( S5 \right) \end{aligned}$$

**Input gate**

$$\begin{aligned} i_{t}=\sigma\left( W_{i}\cdot\left[ h_{t-1},x_{t} \right]+b_{i} \right)\#\left( S6 \right) \end{aligned}$$

$$\begin{aligned} \hat{C}_{t}=\tanh\left( W_{c}\left[ h_{t-1},x_{t} \right]+b_{c} \right)\#\left( S7 \right) \end{aligned}$$

$$\begin{aligned} C_{t}=f_{t}\odot C_{t-1}+i_{t}\odot\tilde{C}_{t}\#\left( S8 \right) \end{aligned}$$

**Output gate**

$$\begin{aligned} O_{t}=\sigma\left( W_{0}\cdot\left[ h_{t-1},x_{t} \right]+b_{0} \right)\#\left( S9 \right) \end{aligned}$$

$$\begin{aligned} h_{t}=O_{t}\odot\tanh\left( C_{t} \right)\#\left( S10 \right) \end{aligned}$$

where *W_f_*, *W_i_*, *W_c_*, *W_o_* are the weights connected with each part and are trainable parameters; *h_t-1_* is the output value at the time of t-1; *x_t_* is the input at time t; *σ* is the Sigmoid activation function; *i_t_* is the degree to which the input information enters the unit state; $\tilde{C}_{t}$ is the new candidate value; *O_t_* is the value of the output gate; *h_t_* is output at the current time. ⊙ is the Hadamard product operation (multiplication by elements).

The motor inertia prediction problem is a regression problem in deep learning. To evaluate the deviation between the predicted value and the real value, the mean square deviation (MSE) function is selected to evaluate the training effect. In order to achieve the best training effect, the network parameters are constantly optimized to reduce the loss function value. The mean square loss function is calculated as follows:

$$\begin{aligned} L_{MSE}\left( y,\hat{y} \right)=\frac{1}{n}\sum_{t=1}^{n} \left( y_{t}-\hat{y}_{t} \right)^{2}\#\left( S11 \right) \end{aligned}$$

In order to reduce the value of the loss function, an optimization algorithm should be adopted. In most cases, the neural network needs to optimize a non-convex function. The most basic optimization algorithm is gradient descent, which requires the use of all sample data and requires too much iterative calculation. With the development of deep learning theory, many other optimization algorithms based on gradient descent have appeared, such as small batch stochastic gradient descent, the momentum method, and RMSProp. The adaptive moment estimation algorithm (Adam) is a more powerful and effective optimization algorithm produced by combining the above algorithms [13,14]. It adjusts the learning rate of each parameter by using the first-order moment estimation and second-order moment estimation of the gradient. After bias correction, the learning rate of each iteration has a definite range to ensure the parameters are relatively stable. The detailed calculation process is given below [15]:

$$\begin{aligned} m_{t}=\alpha m_{t-1}+\left( 1-\alpha\right)\nabla C\left( \omega\right)\#\left( S12 \right) \end{aligned}$$

$$\begin{aligned} v_{t}=\beta v_{t-1}+\left( 1-\beta\right)\nabla C\left( \omega\right)^{2}\#\left( S13 \right) \end{aligned}$$

$$\begin{aligned} \hat{m}_{t}=\frac{m_{t}}{1-\alpha^{t}} \#\left( S14 \right) \end{aligned}$$

$$\begin{aligned} \hat{v}_{t}=\frac{v_{t}}{1-\beta^{t}}\#\left( S15 \right) \end{aligned}$$

$$\begin{aligned} \omega_{t+1}=\omega_{t}-\eta\frac{1}{\sqrt{\hat{v}_{t}}}\hat{m}_{t}\#\left( S16 \right) \end{aligned}$$

where *m_t_* and *v_t_* are momentum parameters; *α* and *β* are non-negative weighted parameters; *ω_t_* is the network parameter; *C(ω)* is the iterative loss; *η* is the learning rate.

Dropout and early stop algorithms are used during model training to prevent overfitting. Dropout sets a fixed probability p in the network and activates neurons, accounting for a 1-p ratio of all neurons each time, which is equivalent to forming a large neural network with countless small neural networks to avoid overfitting the network [16,17]. With the increase in training times, the loss of the model on the training set decreases continuously, while the loss on the verification set may show a U-shaped curve. The early stop mechanism stops the training at the lowest point of the verification loss and saves the model parameters to ensure the generalization ability of the model.

The algorithm flow of LSTM prediction deviation designed in this study is shown in Figure S6, and its specific steps are as follows:

**Step 1**: First, the time series data set is created, the abnormal data in the data is removed, and then the data is normalized to 0~1. The training data is proportionally divided into a training set and a test set, where the training set is used to train the model and the test set provides an initial assessment of the model's capabilities. Finally, the sequence adjustment of the data in the training set and the test set is carried out, respectively, to ensure the temporal relationship within the short series while reducing the temporal relationship between the short series, to ensure the generalization ability of the model and avoid overfitting.

**Step 2**: Pytorch built-in network is used to build an LSTM network, including an LSTM timing prediction layer and a linear regression output layer. A stacked LSTM network is used to increase the depth of the network and improve the nonlinear representation ability of the model.

**Step 3**: The neural network is transplanted into the graphics processing unit (GPU), and the training data is input into the LSTM network for model training using the MSELoss loss function and the Adam optimization algorithm. Each iteration calculates the training loss and validation loss, stops the training when the validation loss does not decrease significantly for several successive iterations, returns the network parameters with the lowest validation loss, and saves them.

**Step 4**: The test data is input into the trained inertia prediction model, and the current inertia prediction value is output.


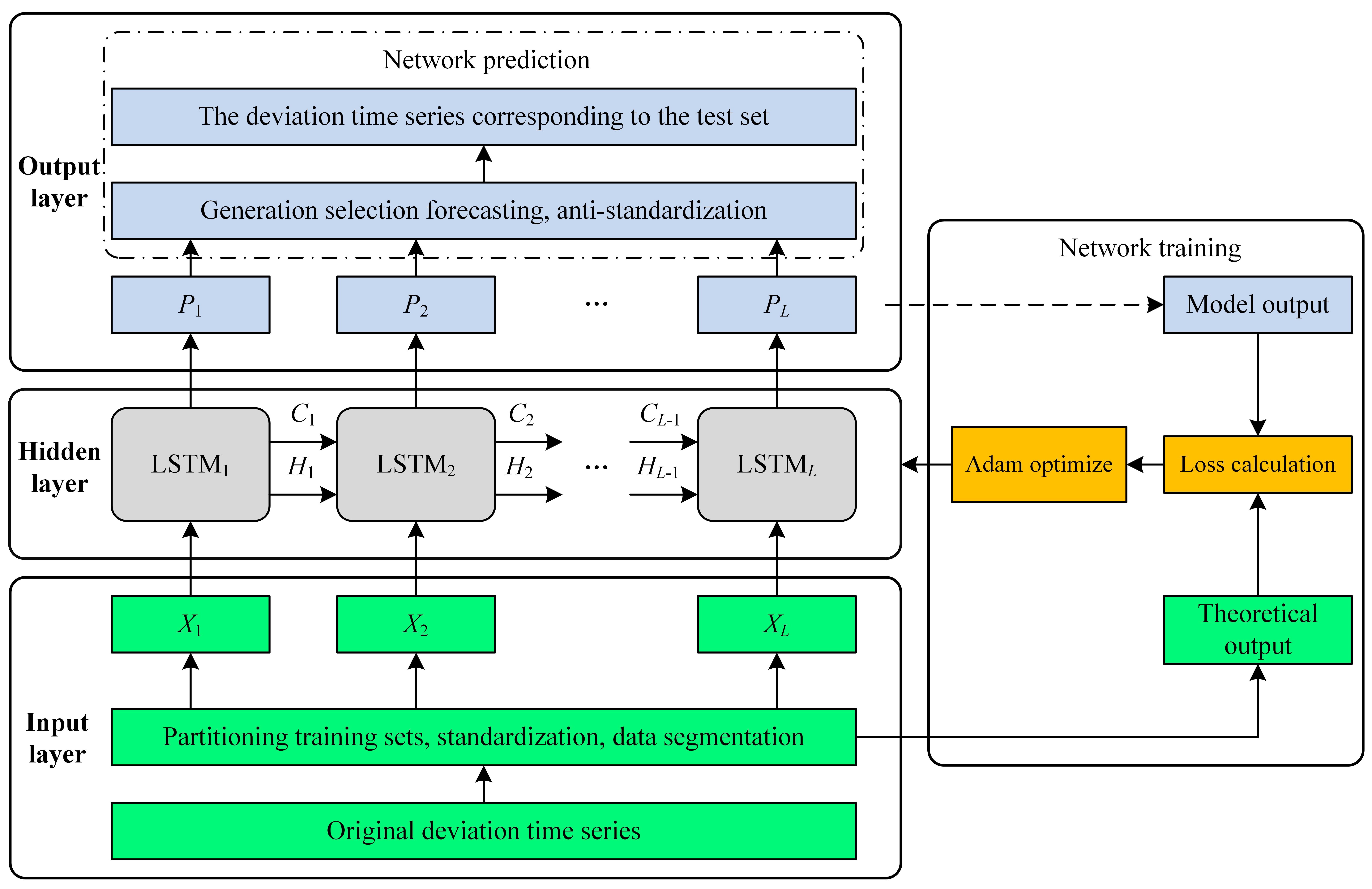


**Figure S6.** LSTM based framework for time series prediction.

5. The main technical parameters of the mechanical structure

**Table S2.** Comparison of technical parameters of one-stage and two-stage pumps.

| **Technical parameter** | **One-stage** | **Two- stage** |
| --- | --- | --- |
| Inside diameter of the reservoir (mm) | 12.4 | 9.59 |
| Gear reduction ratio | 219.488:1 | 739.95:1 |
| Screw pitch (mm) | 0.7 | 0.7 |
| Effective travel of screw (mm) | 24.84 | 41.53 |
| Number of lines of optical code disc | 80 | 640 |

6. Statistical structure and test results of the two-stage pump

**Table S3**. Paired sample statistics

| **Paired** | | **Mean value** | **Number of cases** | **Standard deviation** | **Standard error mean** |
| --- | --- | --- | --- | --- | --- |
| 0.1 U | one-stage pump | 5.7800 | 10 | 0.14386 | 0.04549 |
|  | two-stage pump | 5.6700 | 10 | 0.27301 | 0.08633 |
| 0.2 U | one-stage pump | 5.1400 | 10 | 0.14937 | 0.04723 |
|  | two-stage pump | 4.7800 | 10 | 0.40368 | 0.12765 |
| 0.3 U | one-stage pump | 4.3900 | 10 | 0.49535 | 0.15664 |
|  | two-stage pump | 4.3400 | 10 | 0.44930 | 0.14208 |
| 0.4 U | one-stage pump | 4.5800 | 10 | 0.12671 | 0.04007 |
|  | two-stage pump | 4.5200 | 10 | 0.28547 | 0.09027 |
| 0.5 U | one-stage pump | 4.4500 | 10 | 0.32255 | 0.10200 |
|  | two-stage pump | 4.3500 | 10 | 0.18101 | 0.05724 |
| 0.6 U | one-stage pump | 3.7500 | 10 | 0.37347 | 0.11810 |
|  | two-stage pump | 3.6100 | 10 | 0.41540 | 0.13136 |
| 0.7 U | one-stage pump | 4.3900 | 10 | 0.32738 | 0.10353 |
|  | two-stage pump | 3.8600 | 10 | 0.46636 | 0.14748 |
| 0.8 U | one-stage pump | 3.6600 | 10 | 0.54366 | 0.17192 |
|  | two-stage pump | 3.2700 | 10 | 0.48242 | 0.15255 |
| 0.9 U | one-stage pump | 3.7200 | 10 | 0.35787 | 0.11317 |
|  | two-stage pump | 3.6600 | 10 | 0.37118 | 0.11738 |
| 1.0 U | one-stage pump | 3.6000 | 10 | 0.10669 | 0.03374 |
|  | two-stage pump | 3.2600 | 10 | 0.22100 | 0.06989 |

**Table S4**. Paired sample test table and test difference

| **Paired** | **Mean value** | **Standard deviation** | **Standard**  **error mean** | **Difference 95% confidence**  **interval** | | **t** | **DOF** | **P-value** |
| --- | --- | --- | --- | --- | --- | --- | --- | --- |
|  |  |  |  | Lower limit | Upper  limit |  |  |  |
| 0.1 U | 0.11500 | 0.14339 | 0.04534 | 0.01242 | 0.21758 | 2.536 | 9 | 0.032 |
| 0.2 U | 0.35300 | 0.35923 | 0.11360 | 0.09602 | 0.60998 | 3.107 | 9 | 0.013 |
| 0.3 U | 0.05800 | 0.65270 | 0.20640 | 0.40891 | 0.52491 | 0.281 | 9 | 0.785 |
| 0.4 U | 0.06400 | 0.16140 | 0.05104 | 0.05146 | 0.17946 | 1.254 | 9 | 0.241 |
| 0.5 U | 0.10100 | 0.14836 | 0.04691 | 0.00513 | 0.20713 | 2.453 | 9 | 0.038 |
| 0.6 U | 0.13500 | 0.17024 | 0.05384 | 0.01321 | 0.25679 | 2.508 | 9 | 0.033 |
| 0.7 U | 0.52400 | 0.18963 | 0.05997 | 0.38835 | 0.65965 | 8.738 | 9 | <0.001 |
| 0.8 U | 0.38700 | 0.18172 | 0.05747 | 0.25700 | 0.51700 | 6.734 | 9 | <0.001 |
| 0.9 U | 0.06500 | 0.04353 | 0.01376 | 0.03386 | 0.09614 | 4.723 | 9 | 0.001 |
| 1.0 U | 0.34300 | 0.11700 | 0.03700 | 0.25930 | 0.42670 | 9.270 | 9 | <0.001 |

6. the influence of different hidden layer structures on the prediction accuracy

**Table S5.** Hidden layer mixed structure model comparison.

| **Data type** | **MAPE** | **RMSE** |
| --- | --- | --- |
| DDL | 21.07 | 65.6697 |
| DLD | 28.09 | 68.8779 |
| DLL | 26.60 | 67.8753 |
| LDD | 20.92 | 64.9216 |
| LDL | 20.25 | 59.8416 |
| LLD | 21.13 | 60.0345 |
| LLLD | 23.12 | 68.9113 |
| DLDL | 23.77 | 70.0215 |
| DLLD | 21.76 | 64.4756 |

[1] Malinowski M, Kazmierkowski MP, Trzynadlowski AM. A comparative study of control techniques for PWM rectifiers in AC adjustable speed drives. IEEE Trans. Power Electron. 2003; 18: 1390-1396. https://doi.org/10.1109/TPEL.2003.818871.

[2] Maung MM, Latt MM, Nwe CM, DC Motor Angular Position Control using PID Controller with Friction Compensation, Int. J. Sci. Res. Publ. IJSRP. 2018; 8: 149-155. https://doi.org/10.29322/IJSRP.8.11.2018.p8321.

[3] Somwanshi D, Bundele M, Kumar G, Parashar G. Comparison of Fuzzy-PID and PID Controller for Speed Control of DC Motor using LabVIEW, Procedia Comput. Sci. 2019; 152: 252-260. https://doi.org/10.1016/j.procs.2019.05.019.

[4] Can E, Sayan H. The performance of the DC motor by the PID controlling PWM DC-DC boost converter, Tehnički Glasnik. 2017;11: 182-187. https://avesis.gazi.edu.tr/yayin/e6537b35-36af-4483-b7ba-f3a890c0bf96/the-performance-of-the-dc-motor-by-the-pid-controlling-pwm-dc-dc-boost-converter

[5] Schmidhuber J. Deep learning in neural networks: An overview, Neural Netw. 2015; 61: 85-117. https://doi.org/10.1016/j.neunet.2014.09.003.

[6] Chen J, Zhang Y, Wu J, Cheng W, Zhu Q. SOC estimation for lithium-ion battery using the LSTM-RNN with extended input and constrained output, Energy. 2023; 262: 125375. https://doi.org/10.1016/j.energy.2022.125375.

[7] Dudukcu HV, Taskiran M, Cam Taskiran ZG, Yildirim T. Temporal Convolutional Networks with RNN approach for chaotic time series prediction, Appl. Soft Comput. 2023; 133: 109945. https://doi.org/10.1016/j.asoc.2022.109945.

[8] Van Houdt G, Mosquera C, Nápoles G.A review on the long short-term memory model, Artif. Intell. Rev. 2020; 53: 5929–5955. https://doi.org/10.1007/s10462-020-09838-1.

[9] Xin J, Zhou C, Jiang Y, Tang Q, Yang X, Zhou J. A signal recovery method for bridge monitoring system using TVFEMD and encoder-decoder aided LSTM, Measurement. 2023; 214: 112797. https://doi.org/10.1016/j.measurement.2023.112797.

[10] Zhao L, Li Z, Qu L, Zhang J, Teng B. A hybrid VMD-LSTM/GRU model to predict non-stationary and irregular waves on the east coast of China, Ocean Eng. 2023; 276: 114136. https://doi.org/10.1016/j.oceaneng.2023.114136.

[11] Ming W, Sun P, Zhang Z, Qiu W, Du J, Li X, Zhang Y, Zhang G, Liu K, Wang Y, Guo X. A systematic review of machine learning methods applied to fuel cells in performance evaluation, durability prediction, and application monitoring, Int. J. Hydrog. Energy. 2023; 48(13): 5197-5228. https://doi.org/10.1016/j.ijhydene.2022.10.261

[12] Yu Y, Si X, Hu C, Zhang J. A Review of Recurrent Neural Networks: LSTM Cells and Network Architectures. Neural Comput. 2019;31: 1235-1270. https://doi.org/10.1162/neco_a_01199

[13] Chang Z, Zhang Y, Chen W. Electricity price prediction based on hybrid model of adam optimized LSTM neural network and wavelet transform, Energy. 2019; 187: 115804. https://doi.org/10.1016/j.energy.2019.07.134.

[14] Yadav RK, Anubhav. PSO-GA based hybrid with Adam Optimization for ANN training with application in Medical Diagnosis, Cogn. Syst. Res. 2020; 64: 191-199. https://doi.org/10.1016/j.cogsys.2020.08.011.

[15] Varshney RP, Sharma DK. Optimizing Time-Series forecasting using stacked deep learning framework with enhanced adaptive moment estimation and error correction. Expert Syst. Appl. 2024;249: 123487. https://doi.org/10.1016/j.eswa.2024.123487

[16] Cheng G, Peddinti V, Povey D, Manohar V, Khudanpur S, Yan Y An Exploration of Dropout with LSTMs, in: Interspeech 2017, ISCA: p. 1586-1590. https://doi.org/10.21437/Interspeech.2017-129.

[17] Baldi P, Sadowski P. The dropout learning algorithm, Artif. Intell. 2014; 210: 78-122. https://doi.org/10.1016/j.artint.2014.02.004.
